# A review of horses as a source of spreading livestock-associated methicillin-resistant *Staphylococcus aureus* to human health

**DOI:** 10.14202/vetworld.2022.1906-1915

**Published:** 2022-08-11

**Authors:** Aswin Rafif Khairullah, Sri Agus Sudjarwo, Mustofa Helmi Effendi, Sancaka Chasyer Ramandinianto, Agus Widodo, Katty Hendriana Priscilia Riwu

**Affiliations:** 1 Doctoral Program in Veterinary Science, Faculty of Veterinary Medicine, Universitas Airlangga, Kampus C Unair, Jl. Mulyorejo, Surabaya, Jawa Timur 60115, Indonesia; 2 Department of Veterinary Pharmacology, Faculty of Veterinary Medicine, Universitas Airlangga, Kampus C Unair, Jl. Mulyorejo, Surabaya, Jawa Timur 60115, Indonesia; 3 Department of Veterinary Public Health, Faculty of Veterinary Medicine, Universitas Airlangga, Kampus C Unair, Jl. Mulyorejo, Surabaya, Jawa Timur 60115, Indonesia; 4 Lingkar Satwa Animal Care Clinic, Jl. Sumatera No. 31L, Gubeng, Surabaya, Jawa Timur 60281, Indonesia

**Keywords:** Horse, LA-MRSA, Public health, Risk factors

## Abstract

Livestock-associated methicillin-resistant *Staphylococcus aureus* (LA-MRSA) was first discovered in horses in 1989. Since then, LA-MRSA has begun to be considered an important strain of pathogenic bacteria in horses, which can cause LA-MRSA infection and colonization in humans with public health impacts. The anterior nares are the primary site of LA-MRSA colonization in horses, although LA-MRSA colonization may also occur in the gastrointestinal tract in horses. LA-MRSA-infected horses typically exhibit clinical infection or may not exhibit clinical infection. There are two potential risks associated with LA-MRSA colonization in horses: The possibility of disease development in horses infected with LA-MRSA and the possibility of LA-MRSA transfer to humans and other horses. The diagnosis of LA-MRSA in horses can be made by conducting *in vitro* sensitivity testing for oxacillin and cefoxitin, and then followed by a molecular test using polymerase chain reaction. LA-MRSA transmission in animal hospitals and on farms is most likely due to contact with horses infected or colonized by LA-MRSA. The history of prior antibiotic administration, history of prior LA-MRSA colonization, and length of equine hospitalization were described as risk factors in cases of infection and colonization of LA-MRSA in horses. Nebulized antibiotics may be a viable alternative to use in horses, but nebulized antibiotics are only used in horses that are persistently colonized with LA-MRSA. Controlling the spread of LA-MRSA in horses can be done by regularly washing horses, eradicating vectors in horse stalls such as rats, and maintaining the cleanliness of the stable and animal hospital environment. Meanwhile, cleaning hands, using gloves, and donning protective clothes are ways that humans can prevent the transmission of LA-MRSA when handling horses. This review will explain the definition of LA-MRSA in general, LA-MRSA in horses, the epi­demiology of LA-MRSA in horses, the diagnosis of LA-MRSA in horses, the transmission of LA-MRSA in horses, risk factors for spreading LA-MRSA in horses, public health impact, treatment of LA-MRSA infection in horses, and control of the spread of LA-MRSA in horses.

## Introduction

Methicillin-resistant *Staphylococcus aureus* (MRSA) is a strain of *Staphylococcus aureus* that is resistant to almost all β-lactam antibiotics and is often resistant to antibiotics other than β-lactams [1]. This resistance reaction occurs due to the activity of the penicillin-binding protein encoded by the *mec*A and *mec*C genes located on the Staphylococcal cassette chromosome *mec* (SCC*mec*) [2–5]. MRSA is known globally to spread nosocomially in hospital settings, known as hospital-acquired MRSA (HA-MRSA) [1, 6, 7]. In addition, MRSA that occurs in the community without any association with healthcare facilities is known as community-acquired MRSA (CA-MRSA) [8]. MRSA in animals was first identified in dairy cows with cases of mastitis in 1972 [9], followed by sporadic observations of MRSA infections in various animals, including MRSA infections first identified in horses in 1989 [10], which eventually began to develop into known MRSA that occurs in livestock [11–14] and pets [15, 16] is known as livestock-associated MRSA (LA-MRSA) [17, 18].

Numerous livestock throughout Europe, North America, and Asia have been found to have the LA-MRSA strain with clonal complex 398 (CC398) [19]. Furthermore, since the first LA-MRSA in horses was found in 1989 [8], the recently emerging LA-MRSA CC398 has been identified in equine populations. LA-MRSA is starting to be considered an important pathogenic bacterial strain in horses, which can cause infection and colonization of LA-MRSA in humans [20–26]. Horses that have been infected with LA-MRSA may or may not exhibit clinical infection [27–32]. Skin infections, soft-tissue infections, septic arthritis, bacteremia, osteomyelitis, omphalitis, metritis, and pneumonia are just a few of the clinical diseases that LA-MRSA-infected horses might develop [33]. Since then, LA-MRSA in horses has begun to be considered a public health problem. Although the presence of LA-MRSA in horses has not been widely reported [25, 32, 34], the spread of LA-MRSA in horses is still regarded as a risk to public health [25, 32, 35], so it is important to understand the spread of LA-MRSA in horses so that later control strategies can be implemented to reduce the risk of spreading LA-MRSA to horses in farms and animal hospitals [25, 35, 36].

This review will explain the definition of LA-MRSA in general, LA-MRSA in horses, the epidemiology of LA-MRSA in horses, the diagnosis of LA-MRSA in horses, the transmission of LA-MRSA in horses, risk factors for spreading LA-MRSA in horses, public health impact, treatment of LA-MRSA infection in horses, and control of the spread of LA-MRSA in horses.

## LA-MRSA

LA-MRSA is an opportunistic strain of bacteria that can be identified in humans, livestock, and pets. In 1972, the first LA-MRSA case in livestock was a subclinical mastitis case in dairy cattle in Belgium, in which the LA-MRSA was of human origin [9]. Since then, LA-MRSA in livestock has been reported frequently and has even begun to be found in domestic animals [37]. Ceballos *et al*. [38] identified a new LA-MRSA lineage, the type (ST) 398 sequence that is in the CC398 grouping, which is now often found in livestock and domestic animals and infects humans. Thus, LA-MRSA-associated farm and domesticated animals with the CC398 clonal complex are starting to be widely reported globally [39], in horses [40], cattle [41], poultry [42], dogs [43], and cats [44].

## LA-MRSA in Horse

In 1989, LA-MRSA was first identified in a mare with metritis in Japan [10]. Since then, several investigators have identified the presence of LA-MRSA among horses in America, Europe, and Asia, with slight differences in the prevalence rates between regions [21, 43, 45–49]. In several cases of LA-MRSA infection in horses, clones that differ from those found in normal livestock and domestic animals have been identified, namely, the CC8 clonal complex, which was previously associated with HA-MRSA infection in the hospital [21, 47, 48, 50].

There are still few reports of LA-MRSA infection in horses. In 1997, Hartman *et al*. [51] reported that there was LA-MRSA infection in postoperative horses in the hospital, but the source of the LA-MRSA infection is still unknown. Following the discovery of LA-MRSA in 11 horses for 13 months at a veterinary hospital in Michigan, the USA, in 1999 [52], three medical personnel at the hospital were identified as infected with LA-MRSA originating from these horses. Over the past few years, several LA-MRSA infections have been reported in horses with wound infections, surgical site infections, pneumonia, arthritis, osteomyelitis pneumonia, metritis, catheter site infections, and dermatitis [53, 54].

Not all LA-MRSA infections in horses cause clinical infection. There are two potential risks of LA-MRSA colonization in horses; namely, there is a potential for disease development in horses infected with LA-MRSA and the potential for transmission of LA-MRSA transmission to other horses and humans. Moremi *et al*. [55] found that up to 29% of human patients infected with MRSA on discharge from the hospital developed subsequent MRSA infections, including bacteremia, osteomyelitis, septic arthritis, and pneumonia but this did not occur in cases of infection. LA-MRSA in horses, which is a clinical symptom in horses due to LA-MRSA infection only days to weeks after the presence of LA-MRSA in horses is discovered [56]. With this, the case of LA-MRSA infection in horses should be considered because it is thought to put the horse at risk of disease progression. The most common sites for MRSA colonization in humans the nasal cavity, hair, nails, skin, vaginal axilla, and perineum. In contrast, the most common sites for MRSA colonization in horses are the nasal cavity [57].

In Europe, apart from LA-MRSA ST398 clones, CA-MRSA ST1 and CA-MRSA ST254 clones have also been identified in horses [25, 58, 59]. In a study conducted in England, only three horses out of 152 horses were identified with MRSA; the MRSA strains were included in clones of LA-MRSA CC398, HA-MRSA CC8, and HA-MRSA CC22 [53]. In Germany, the transition from a human-associated CA-MRSA clone to an LA-MRSA CC398 clone was reported in 2006 [60]. In a study conducted in Switzerland, changes in the distribution of LA-MRSA clones in horses included LA-MRSA strains collected from 2005 to 2011, whereas LA-MRSA ST398 was found in 2007 [26]. Risk factors for LA-MRSA infection in horses can come from the use of topical antibiotics, use of systemic glucocorticoids, and visits by a veterinarian [33]; this fact emphasizes that LA-MRSA infection in horses is associated with antibiotic use, immunosuppression, and hospitalization.

## Epidemiology LA-MRSA in Horse

The epidemiology of LA-MRSA in horses has not been widely reported [61]. The anterior nares are the primary site of LA-MRSA colonization in horses, although LA-MRSA colonization may also occur in the gastrointestinal tract in horses [62]. Approximately 10% of the nasal cavities of healthy horses have LA-MRSA [63]. Within the reservoir of infected horses, LA-MRSA is likely to spread among the equine population [64]. LA-MRSA colonization rates are likely to vary widely among horse populations and different geographic areas, as much as 0–5% of the equine population has been examined in different regions, with the LA-MRSA prevalence rate in certain horse populations approaching 50% [65]. Although LA-MRSA colonization is usually transient in horses, LA-MRSA is easily transmitted among horse populations and is likely to continue to spread within equine populations [31]. Even though the majority of infected horses did not exhibit clinical illness, LA-MRSA colonization was a risk factor for disease cases in horses that were being treated by veterinarians in hospitals [31]. Horses infected with LA-MRSA are at risk of transmitting LA-MRSA to other horses and humans [66].

LA-MRSA infection in horses can occur sporadically or in outbreaks on farms and animal hospitals [19]. Horses of all ages can become infected with LA-MRSA, including foals that are <24-h-old [65]. The predisposition of horse age, sex, and race association with LA-MRSA infection in horses has not been reported [65]. LA-MRSA infection cases appear to occur more frequently in horse farms than in hospital animals, although there is still a lack of objective evidence of an increased risk of LA-MRSA transmission in horses [63]. Several risk factor analyses for LA-MRSA transmission in horses have been detailed, including the previous history of LA-MRSA colonization in horses, the previous discovery of LA-MRSA colonies in horses, antibiotic administration for the previous 30 days, and neonatal critical care in a veterinary hospital [66]. Administration of aminoglycosides and cephalosporins during hospitalization was associated with the rate of LA-MRSA colonization in horses [66].

In 1997, Hartmann *et al*. [51] reported one LA-MRSA strain isolated from postoperative wound infection in horses in the United States, whereas in 1999, Seguin *et al*. [52] reported a case of LA-MRSA infection in horses at the University of Michigan veterinary hospital in the United States. Depending on the region being examined, different forms of diseases in horses are caused by LA-MRSA colonization and infection. The Canadian MRSA-5 strain is seldom detected in people, while it is most frequently isolated in horses and horse workers in Canada [63]. In Europe, other LA-MRSA strains have been found in horses, including LA-MRSA ST398, in which the LA-MRSA CC398 strain is a strain of pig origin [58, 67]. In epidemiological and genome analytic studies, LA-MRSA ST398 was classified into two distinct phylogenetic clades, namely the livestock clade and the basal human clade [68, 69]. The main characteristics that lie in the preceding clade are the disappearance of the bacteriophage FSa3 and the acquisition of a Tn916-like transposon carrying the *tet*M-encoding gene, LA-MRSA ST398 has a human-specific immune avoidance cluster gene carrying *chp* and *scn*, encoding a chemotaxis inhibitor protein and a complement inhibitor Staphylococcus respectively [68, 69].

In certain cases, horses that have been infected by LA-MRSA have clones that are different from the clones found in livestock and domestic animals, but have the same clones as MRSA in humans; for example, CC8 clones (ST8 and ST254) were discovered in horses; these clones are HA-MRSA clones that are frequently detected in hospitals in Canada, but clones ST1, ST22, and ST254 that are also HA-MRSA clones have also been discovered among horse isolates in Europe [58]. Studies conducted in Canada and Europe have reported that the LA-MRSA clone that frequently infects horses is LA-MRSA CC398 (ST398) [31, 58, 60].

There are increasing reports of LA-MRSA’s ability to spread among horse populations and communities, particularly in humans working on horse farms and animal hospitals. Farmers who had contact with foals infected with LA-MRSA ST8 (USA500) showed symptoms of a skin infection caused by the LA-MRSA strain in the foal [22]. Staff workers at veterinary hospitals and veterinarians are at a higher risk of contracting LA-MRSA from horses as long as cases of LA-MRSA infection occur in horses in veterinary hospitals [70]. However, LA-MRSA CC398 in horses is likely a specific clade of LA-MRSA occurring in general livestock, which does not spread beyond animal hospitals and horse farms [71].

## Diagnosis of LA-MRSA in Horse

The diagnosis of LA-MRSA infection requires the isolation of *S. aureus* from the infected site and testing for methicillin resistance [72]. *In vitro* sensitivity to oxacillin is often used as a benchmark for methicillin resistance because oxacillin is more stable in use *in vitro* [73]. *S. aureus* strains resistant to oxacillin were considered resistant to methicillin, but false-positive and false-negative test results were obtained [74]. False-positive test results sometimes occur because LA-MRSA strains produce high levels of β-lactamase, which can lead to low levels of oxacillin resistance [75]. Such LA-MRSA strains can be identified by reviewing the sensitivity of other antibiotics because they may be sensitive to cephalosporins or penicillin-anti-β-lactamase combinations such as amoxicillin-clavulanic acid [76]. Any LA-MRSA isolate suspected to be sensitive to a combination of β-lactamase inhibitors or other β-lactam antibiotics needs further testing [77]. False-negative test results can be obtained due to incomplete expression of the *mec*A or *mec*C coding genes because oxacillin can be bad *in vitro* inducer of the *mec*A or *mec*C gene encoding genes [78]. Other antibiotics, such as cefoxitin, can also be used in *in vitro* sensitivity testing as a benchmark for methicillin resistance because cefoxitin has a greater ability to induce a β-lactam antibiotic resistance phenotype [79]. Isolates suspected of being LA-MRSA should be further tested to detect the *mec*A or *mec*C coding gene by polymerase chain reaction [64] or for PBP2a by latex agglutination assay [80].

In some situations, screening in clinically healthy horses to identify LA-MRSA colonization may be indicated [64]. The culture method taken from horse nose swabs appears to be the optimal and frequently used method [81]. Samples were collected by inserting a 10–15 cm nasal swab into the horse’s nasal cavity and letting the nasal swab touch the nasal mucosa on withdrawal [81]. Several types of culture media are used to detect LA-MRSA differential and selectively [73]. The use of enrichment media can increase the sensitivity of the test, but the drawback of using enrichment media is that it takes an incubation time of 24 h before results can be available [82]. In horses, molecular testing using real-time polymerase chain reaction of nasal swab specimens was used for LA-MRSA identification [83].

## Transmission LA-MRSA in Horse

MRSA is a health problem of worldwide concern. Approximately 30% of the general population has been infected with *S. aureus* and a small proportion of these *S. aureus* isolates are MRSA [84, 85]. However, hospital and farm environments have a higher proportion of LA-MRSA transmission; although there are different proportions in each country, people who act as health workers, veterinarians, and breeders will be more at risk of getting LA-MRSA [64, 86].

Healthy horses usually have a low LA-MRSA prevalence rate, compared to horses that are currently being treated in a veterinary hospital will have a higher LA-MRSA prevalence rate [87, 88] and nosocomial LA-MRSA infections in horses may cause serious, even fatal [89], cases of clinical infection in horses due to LA-MRSA colonization. However, the high prevalence of LA-MRSA in animal hospitals and farms poses a risk not only to horses but also to health workers, breeders, and veterinarians [90, 91]. In recent years, LA-MRSA in horses has been the subject of several scientific studies. Kuroda *et al*. [92] reported that horses in healthy conditions were rarely a source of LA-MRSA transmission; in addition, veterinarians who had been infected with LA-MRSA from other patients could be a source of LA-MRSA transmission in horses.

Nosocomial transmission of MRSA in the hospital is likely to occur through the hands of hospital staff contaminated with MRSA as a result of contact with patients infected or colonized by MRSA [93]. Hospital staff infected and colonized with MRSA can also transmit MRSA [93]. LA-MRSA transmission in veterinary hospitals and on farms is likely to occur in the same way as a result of contact with animals infected or colonized by LA-MRSA. The transmission of LA-MRSA transmission from veterinary and livestock hospital environments has not been well documented, although LA-MRSA contamination originating from animal hospitals and farms has been reported [64, 94, 95]. LA-MRSA isolates were most often isolated from areas that had frequent contact with the horse’s nasal cavity such as stable walls, hay, feed bins, and water buckets [96]. The environment of animal hospitals and farms is not considered an important source of LA-MRSA transmission, but the environment of animal hospitals and farms can be a source of LA-MRSA transmission if not properly managed [87]. Therefore, it is very important to maintain a clean environment in veterinary hospitals and farms, especially in areas that frequently come into contact with horse noses [96, 97].

## Risk Factor for Spreading LA-MRSA in Horse

LA-MRSA infection and colonization is a serious condition that can occur in horses as well as in humans. The risk factors reported in cases of MRSA infection and colonization in humans were history of antibiotic administration, contact with hospital staff, history of previous hospitalization, and hospital overcrowding [57, 98–101]. Meanwhile, the reported risk factors for LA-MRSA infection and colonization in horses have not been adequately evaluated [102].

Horses identified as LA-MRSA positive were more likely to have clinical LA-MRSA infection than horses that were identified as LA-MRSA negative [21]. LA-MRSA clones found in the nasal cavity of horses will still be found in cases of subsequent infection [25]. The administration of aminoglycosides and ceftiofur antibiotics is one of the risk factors that cause horses to become LA-MRSA-positive during treatment [21]. Examples of other antibiotics that are risk factors for LA-MRSA transmission are fluoroquinolone exposure [103] and the causal relationship between antibiotic use and LA-MRSA infection [104]. Horses from farms with positive LA-MRSA testing, horses from those farms, LA-MRSA contamination during hospitalization, and LA-MRSA contamination during surgery in a veterinary facility have all been described as risk factors in horses that have been identified as LA-MRSA positive [66]. A significant association between surgical incision scars and LA-MRSA nosocomial infection has also been reported [33].

Increased risk factors for veterinarians and veterinarians infected with LA-MRSA and the potential for further spread of LA-MRSA should be considered [25, 58, 70, 105]. In Dutch veterinary hospitals, the same type of *spa*, t011, and t2123 were identified in horses and animal hospital staff infected with LA-MRSA [25]. In a study conducted in an Israeli hospital, 12 horses out of 84 horses (14.3%) and 16 of 139 personnel (11.5%) were identified as having LA-MRSA ST5, a type of *spa* t535 [70]. In addition, there is a greater risk of LA-MRSA transmission to veterinarians, animal hospital staff, breeders, and people living near animal hospitals and farms [70].

## Public Health Impact

The role of horses as a means of transportation and recreation can be a risk of colonization and LA-MRSA infection in humans [22]. In a study conducted in Denmark, it was found that practicing veterinarians were substantially more likely to be infected with LA-MRSA than those who had no contact with horses; exposure to horses was thought to increase the likelihood of LA-MRSA transmission [106]. Veterinary professionals attending international equine conferences have also been shown to have LA-MRSA colonization [105]. The care of horses that tested positive for LA-MRSA or had a prior year history of LA-MRSA infection was linked to an elevated risk of LA-MRSA colonization and infection [107].

Several studies [25, 47, 49, 52, 58] have reported that LA-MRSA isolates from horses and people working on horse farms differ from MRSA isolates in the general population. In Austria LA-MRSA ST254, *spa* type t036 and SCC*mec* type IV were mostly found in horses, followed by LA-MRSA ST398 *spa* type t011, SCC*mec* type IV, and LA-MRSA ST1 *spa* type t127, SCC*mec* type IV [58]. According to the PFGE classification as Canadian MRSA-5, the majority of LA-MRSA isolates reported in horses in Canada were LA-MRSA ST8 *spa* type t008 and SCC*mec* type IV. This suggests that the LA-MRSA clone discovered in humans originated from horses [32]. The LA-MRSA ST398 *spa* type t011 and LA-MRSA ST8 *spa* type t064 were discovered in horses in the Netherlands [25]. From 2000 to 2002, a total of ten horse farms and 27 people in Canadian veterinary hospitals were identified by LA-MRSA [20]. In another study conducted by Schulz *et al*. [94], there was one person, a veterinarian, who had a clinical infection and had the same type of LA-MRSA as the two horses were treated by him [94]. Three individuals who work on horse breeding farms have also reported experiencing skin illnesses caused by LA-MRSA in humans. LA-MRSA isolation tests have revealed that these three individuals had the same strain of LA-MRSA as the foals raised on these farms [22]. The results of screening tests on personnel during cases of LA-MRSA infection in veterinary hospitals indicate that people who have close contact with horses are more likely to be infected with LA-MRSA than people who do not come into contact with horses [25].

## Treatment of LA-MRSA Infection in the Horse

Eradication of colonization and LA-MRSA infection are desirable steps to reduce the likelihood of developing clinical infectious diseases and reduce the risk of further transmission to horses and humans [45]. However, measures to eradicate LA-MRSA colonization and infection have not been evaluated [108].

LA-MRSA colonization and infection are transient in the majority of adult horses and in most horses, the level of LA-MRSA colonization can be eliminated within few weeks of LA-MRSA infection, provided measures are taken to prevent reinfection of horses and other humans [45]. A minority of horses may continue to colonize LA-MRSA for months or even years after being infected with LA-MRSA [94]. In addition, the LA-MRSA infection control protocol may not be feasible in some situations where horse movement is indispensable for training, breeding, competition, or sales [109]. In this situation, antibiotic therapy to eradicate LA-MRSA colonization and infection is necessary; however, antibiotic therapy should not be underestimated because of the speed with which LA-MRSA isolates develop resistance and the limited choice of antibiotics available [110]. Antibiotic therapy may be necessary for persistent LA-MRSA infection or where recommendations for colonization control and LA-MRSA infection are unlikely to be followed [110].

Mupirocin antibiotic therapy administered intravenously is frequently coupled with other antibiotics, such as fusidic acid, to treat colonization, and MRSA infections in people [111]. The use of the topical intranasal antibiotic mupirocin has not been evaluated in horses, but the potential efficacy of this antibiotic therapy is questionable because of the difficulty of administering daily topical antibiotic therapy throughout the horse’s nasal cavity [112]. Oral and parenteral antibiotics can be used for horses; however, most LA-MRSA isolates in horses are resistant to the most commonly used class of antibiotics, and eradication therapy for colonization and LA-MRSA infection will involve the use of the most appropriate antibiotics provided for the treatment of ongoing clinical or equine infections continuously undergoing LA-MRSA colonization [110]. Antibiotic nebulization or inhaler administration of antibiotics may be viable alternatives in horses, but nebulized antibiotics are only used in horses that are persistently colonized with LA-MRSA [113]. Nebulized amikacin (1750 mg in 21 mL total volume) or nebulized enrofloxacin (175 mg in 10 mL total volume) has begun to be used in horses that are continuously colonizing LA-MRSA with shown success in eradicating LA-MRSA colonization [113]. In addition, antibiotic nebulation is not appropriate in cases of LA-MRSA colonization that occurs in the gastrointestinal tract and guttural pockets in horses [114]. Therefore, the efficacy and safety of nebulized antibiotics still require further research.

## Controlling the Spread of LA-MRSA in Horse

Dissemination of horse-derived LA-MRSA in animal hospitals and farms has been reported [25, 94]. Hand hygiene is rated as the most important step in the prevention of LA-MRSA infection nosocomially in humans [115]. LA-MRSA-contaminated hands will be the next major route of LA-MRSA transmission in animal and livestock hospitals [116].

The use of gloves while handling horses and, if necessary wearing protective clothing when handling a horse with a wound infection is basic hygienic precautions for people, breeders, and veterinarians, but they must be followed consistently and firmly [117]. Differences in glove material against LA-MRSA transmission and LA-MRSA transmission using dry gloves have been reported [118]. Nitrile gloves showed the lowest LA-MRSA transmission rate [118], but the absorption of tested body fluids increased LA-MRSA transmission significantly for all glove types. Therefore, it is important to change gloves regularly when handling horses, especially when operating on horses.

Horse mucus scattered in stables and animal hospitals could potentially be a source of LA-MRSA transmission, based on the fact that LA-MRSA has been detected on cell phones, twitches, horse snouts, floors, and medical equipment [94, 119]. In addition, LA-MRSA has also been detected on computer keyboards in veterinary clinics [120]; therefore, it is necessary to maintain the cleanliness of cages and veterinary hospitals to prevent the spread of LA-MRSA.

Bathing horses regularly are also the right step in tackling the spread of LA-MRSA, because it can also be an effort to clean the dust on the horse’s skin surface that has the potential to carry LA-MRSA, the dust on horsehair is indeed difficult to avoid, so it is necessary to bathe the horse with care [121]. It also shows that humans infected with MRSA can spread MRSA through the air [122]. Foot hygiene should also be considered, as LA-MRSA can survive on dry surfaces for months [123].

Nosocomial spread of LA-MRSA in horses during hospitalization has been reported in previous studies [21, 25, 70]. This suggests that horses infected with LA-MRSA should be isolated so that the infection does not transmit LA-MRSA to other patients; in addition, strict isolation measures need to be implemented for horses that have been infected with LA-MRSA [124]. A measure to prevent the spread of LA-MRSA for veterinarians is to wear protective clothing, hat, boots, and gloves before entering the stable of horses that are being isolated [96].

Vector control must also be considered, as was the case in cattle and pig farms where LA-MRSA ST 398 was discovered, where LA-MRSA was also isolated from mice found in cattle and pig pens [125]. Whereas in a horse stable, several vectors are usually found, such as rats, birds, and other pests.

## Conclusion

The LA-MRSA strain can be a bacterial pathogen that can infect horses. LA-MRSA colonization can usually be identified in the nasal cavity of horses. Horses that have been infected with LA-MRSA usually do not show clinical infection; however, there have been some cases that have shown clinical infection. LA-MRSA transmission in animal hospitals and on farms most likely occurs as a result of contact with horses infected or colonized by LA-MRSA. Floor surfaces and objects that frequently come into contact with the horse’s nose are also common sources of LA-MRSA transmission. People who are at risk of getting LA-MRSA from horses are people who work on horse farms, veterinarians, and veterinarians. The spread of LA-MRSA can be controlled by maintaining the cleanliness of the enclosure environment and animal hospital.

## Authors’ Contributions

ARK: Collected samples, analyzed data, and drafted the manuscript. SCR, AW, and KHPR: Designed the study and guidance. SAS and MHE: Revised the manuscript. All authors have read and approved the final manuscript.
